# *Clostridioides difficile* recurrence in individuals with and without cancer: a Swedish population-based cohort study

**DOI:** 10.1007/s15010-024-02193-1

**Published:** 2024-02-26

**Authors:** Peace Mpakaniye, Annelies Boven, Steven Callens, Lars Engstrand, Erika Vlieghe, Nele Brusselaers

**Affiliations:** 1 Centre for Translational Microbiome Research, Department Microbiology, Tumor and Cell Biology, Karolinska Institutet, Stockholm, Sweden; 2 Global Health Institute, Department of Family Medicine and Population Health, University of Antwerp, Antwerp, Belgium; 3 The Vaccine & Infectious Disease Institute, University of Antwerp, Antwerp, Belgium; 4 General Internal Medicine and Infectious Diseases, Ghent University, Ghent University Hospital, Ghent, Belgium; 5 Infectious Diseases, Department of General Medicine, Antwerp University Hospital, Antwerp, Belgium; 6 Department of Public Health and Primary Care, Ghent University, Ghent, Belgium

**Keywords:** Cancer, *Clostridioides difficile*, Recurrence, Real World Evidence, Epidemiology, Risk factors

## Abstract

**Purpose:**

Patients with cancer are vulnerable to *Clostridioides difficile* infection (CDI) due to their disease, treatment and regular hospital contact, yet if CDI-recurrence is more common remains unclear, and differences among cancer types remain unexplored.

**Methods:**

This Swedish nationwide population-based cohort included all 43,150 individuals with recorded CDI (2006–2019) to assess CDI-recurrence in individuals with and without cancer, with binary multivariable logistic regression, stratified by anatomical location, and survival status.

**Results:**

Compared to those without cancer (*N* = 29,543), ongoing cancer (diagnosis < 12 months; *N* = 3,882) was associated with reduced recurrence (OR = 0.81, 95% CI 0.73–0.89), while there was no association with cancer history (diagnosis ≥ 12 months; *N* = 9,725). There was an increased 8-week all-cause mortality (Ongoing cancer: OR = 1.58, 95% CI 1.43–1.74; Cancer history: OR = 1.45, 95% CI 1.36–1.55) compared to those without cancer. Among CDI-survivors, those with ongoing cancer presented with a decreased odds of recurrence (OR = 0.84, 95% CI 0.76–0.94), compared to those without cancer history, with no association for those with cancer history (OR = 1.04, 95% CI 0.97–1.1). Large variations were seen across cancer types, with the highest observed proportion of recurrence in oral and mesothelial cancer, and the lowest for esophageal cancer, although no statistically significant OR were found.

**Conclusion:**

The population-based study indicates that individuals with cancer may have fewerrecurrences than expected, yet variations by cancer type were large, and mortality was high.

**Supplementary Information:**

The online version contains supplementary material available at 10.1007/s15010-024-02193-1.

## Introduction

*Clostridioides* *difficile* is a spore-forming bacterium that can be a member of the intestinal microbiome in healthy individuals [1, 2]. Yet, certain strains may release toxins, making them pathogenic [3, 4]. The human gut microbiome, comprising of bacteria, archaea, viruses, and eukaryotes, can prevent colonization and overgrowth, through direct and indirect mechanisms [1, 2, 5]. However, intestinal dysbiosis, also described as an “unhealthy” alteration of the gut microbiome, is a growing concern [1, 2, 4, 6]. *C. difficile* can contribute to this dysregulated gut microbiome, which may trigger the immune system and generate an environment favorable to overgrowth, colonization and infection [2]. In this case, the combination of ingestion of spores from the toxin-producing *C. difficile*, e.g., during hospitalization, and increased susceptibility (e.g., due to treatment with broad-spectrum antibiotics) can lead to *C. difficile* infection (CDI) [4]. CDI can lead to serious complications such as intense diarrhea, which can cause imbalances in electrolytes, dehydration, unstable blood flow, toxic megacolon, shock, and even mortality [7].

The incidence of CDI in adults and children remains high and is even increasing in several settings [8–11]. As one of the most common healthcare-associated infections in the Western world, CDI is a major health burden and public health threat [7, 8, 12, 13]. According to an extensive meta-analysis including 57 studies on recurrence, approximately 10–20% experience recurrence particularly among hospital-acquired CDI, with a maximum recurrence of 64% [9]. Significant risk factors for CDI recurrence, based on three meta-analyzes, include antibiotic use, advanced age, proton pump inhibitor (PPI) use [14–16], and renal insufficiency [14]. Several meta-analyzes confirmed the effect of antibiotics and PPIs in more detail confirming the increased CDI risk [17–20] and recurrence risk [21–23]. Hospitalization and gut dysbiosis are also reported as risk factors for recurrence but results are inconsistent [6, 9, 16].

Patients with cancer are considered particularly vulnerable to CDI, mainly because of their immunocompromised state, intense treatment, regular healthcare-contact, and higher overall susceptibility to infections [24–26]. Several studies reported a higher incidence of CDI in patients with cancer than those without [27]. Whether this is also the case for recurrence seems less clear, as some studies do report cancer as a risk factor [28, 29], while others do not find significant associations, and effect estimated for cancer as risk factor which are clearly smaller than those related to exposure to antibiotics and PPIs [30–33]. Exploring the risk of recurrence among different cancer types is challenging due to the heterogeneity of cancer types and treatments, yet it is of high clinical relevance, as some individuals may benefit from closer follow-up and adapted treatment if considered at high risk. If there are differences among cancer types, this could shed light, or at least open the discussion on potential pathophysiological mechanisms of CDI and recurrence, particularly to distinguish between re-infection and relapse [34, 35].

There is a lack of large-scale population studies that have investigated the risk of recurrence including community-acquired CDI, comparing all cancer types. Therefore, we conducted a large-scale Swedish population-based study, to investigate CDI recurrence in individuals with cancer (diagnosed within 1 year, and history of cancer) compared to individuals without cancer, and to examine which cancer types (by anatomical location) present with the highest recurrence risk.

## Methods

### Study design

The study is an observational population-based cohort study based on the following high-quality Swedish registries: Patient Registry (In-patient and Specialist Outpatient Registries from 1997 and onwards, when ICD-10 was introduced), Prescribed Drug Registry (initiated July 2005, only out-patient prescribed and dispensed drugs), Death Registry (established in 1952) and Cancer Registry (established in 1958) [36–39]. These data were linked by the individual’s personal identification number which is assigned to each Swedish resident.

This study is based on a larger cohort including all individuals with a CDI diagnosis (*N* = 43,150) in Sweden between January 2006-December 2019, as described earlier [40–42]. Ethical approval was obtained from the National Ethics Committee (2020–02454).

CDI was defined by International statistical Classification of Disease and related health problems 10th revision (ICD-10) code A04.7. Everyone was followed up from the first CDI episode recorded during the study, to death, recurrence or end of the follow-up period of 8 weeks as a CDI infection after 8 weeks is not considered a recurrence but a re-infection.

### Exposure: cancer history

All individuals were categorized in 3 mutually exclusive groups regarding their history of cancer: those with any ongoing cancer (diagnosed < 1 year before the CDI diagnosis), those with cancer history (≥ 1 year before CDI diagnosis) and no ongoing cancer, and the reference group including all without any history of cancer. We chose this 1-year cut-off to distinguish between individuals with likely ongoing cancer treatment/active disease, and those more likely to have successfully finished the initial treatment, although we acknowledge large differences between different cancer types. According to a recent study, multimodal treatment with curative intent of common cancer types often lasts ± 6 months [43].

Primary cancer was defined as the first episode of a malignant cancer using ICD-10 (Supplementary Table 1). We categorized these as oral-, gastrointestinal tract-, respiratory organs-, skin-, mesothelial-, breast-, female genital organs-, male genital organs-, urinary tract-, nervous system-, and other cancer (including cancer types with heterogenous/unclear locations or too low prevalence). Gastro-intestinal cancers were subdivided in esophageal-, stomach-, colorectal-, liver and biliary tract-, pancreas-, and other gastrointestinal cancer-subtypes. Non-melanoma skin cancer (C44) was excluded as it is often underreported, commonly treated in primary care and usually non-fatal. We also excluded the in-situ neoplasms (D00-D09), benign neoplasms (D10-D36) and neoplasms of uncertain or unknown behavior (D37-D48).

### Outcome: CDI recurrence

The main outcome, i.e., CDI recurrence (no recurrence/recurrence), based on discharge diagnosis or specialist outpatient clinic records, was defined as second CDI diagnosis within the first 8 weeks after the primary CDI diagnosis [34, 35]. In addition, the secondary, competing outcome, mortality (alive/death), was defined as death within 8 weeks after primary CDI diagnosis.

### Covariates 

We assessed the following potential confounders: age in years (0–64,65–84 and ≥ 85), sex (Male/Female), chronic comorbidities (Charlson comorbidity score [44, 45], in six categories based on ICD-10 coding, and therefore includes all diagnoses recorded since 1997) and prescribed out-patient drug use during the 180 days prior CDI infection [46], based on the Anatomical Therapeutic Chemical (ATC) classification system (Supplementary Table 1), including antibiotic use (yes/no), aspirin use (yes/no), H2RA use (yes/no), PPI use (yes/no), and non-steroidal anti-inflammatory drug (NSAID) use (yes/no). Origin of CDI was defined as a CDI diagnosis during or within 4 weeks after latest in-hospital admission (hospital-acquired), more than 12 weeks after latest hospital admission (community-acquired) and between 4 and 12 after latest hospital admission (unknown origin) [40, 41].

### Statistical methods

We used binary logistic regression to study the impact of ongoing cancer and cancer history on the odds of CDI recurrence within the time frame of 8 weeks, adjusting for age, sex, origin of CDI, chronic comorbidities, antibiotic use, aspirin use, H2RA use, PPI use, NSAID use, and using all individuals without cancer history as reference. We presented results as odds ratios (OR) and 95% confidence intervals (CI).

As death is a competing risk, a sensitivity analysis was performed, using a similar approach as the main analysis, on individuals who survived at least 8 weeks after the first CDI episode. Additional analyzes were performed to assess the impact of ongoing cancer and cancer history on mortality, adjusting for the same confounders as in the main analysis. To assess CDI recurrence across various cancer types, a stratification analysis was conducted.

Furthermore, we used multivariable cause-specific hazards regression to study the association between ongoing cancer and cancer history on the hazard of CDI recurrence (within 8 weeks), taking into account death as competing risk, with results presented as hazard ratios (HR) and 95% CI [47–49]. To further explore this, acause-specific cumulative incidence function (CIF) was tailored to our study’s competing risks data. This figure provides the absolute risk of experiencing the event CDI recurrence by a given time. Importantly, the CIF accounts for the presence of death as competing risk, with distinct blue lines delineating the cumulative incidence of 'recurrence' and the red lines competing risk of 'death.' This figure not only highlights the individual risk trajectories for 'recurrence' and 'death' but also underscores the interplay between these competing events over time.

Data management and analyzes were performed using Stata version 14.2 and R Studio version 4.2.2.

## Results

**Fig. 1:**
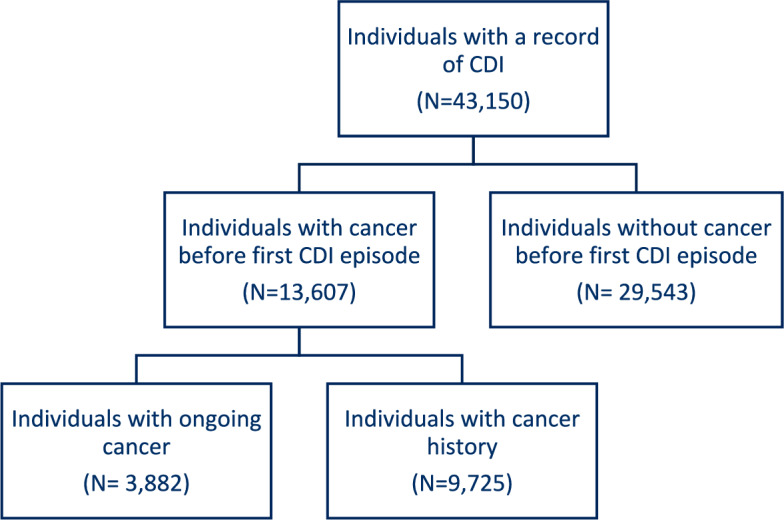
Flow chart of all individuals with a recorded *Clostridioides difficile* infection (CDI) diagnosis in Sweden between 2006 and 2019

The cohort included 43,150 individuals, with 3882 having ongoing cancer, 9725 with a cancer history and 29,543 having no cancer history (Fig. 1). Overall, 45.8% of the individuals were men, while 54.2% were women. Most individuals fell into the 65–84 age group (49.4%), followed by the ≥ 85 age group (25.4%) and the 0–64 age group (25.2%). The Charlson comorbidity score showed a varied health profile, with notable proportions having scores of 2 (18.7%) and 5 (26.7%). Most individuals acquired CDI in a hospital setting (91.6%), while 7.2% was community-acquired. Overall, most individuals had a recent history of antibiotic usage (97.1%) and a substantial portion had used proton pump inhibitors (71.5%) and non-steroidal anti-inflammatory drugs (59.0%) with limited differences between groups. The death rate before CDI recurrence within 8 weeks was 15.4%. The CDI recurrence within 8 weeks was 16.8% (Table 1).

**Table 1:** Descriptive baseline characteristics of all individuals with a *Clostridioides difficile* infection (CDI) in Sweden (2006–2019)

	No cancer history (*N* = 29,543)	Cancer history (*N* = 9725)	Ongoing cancer (*N* = 3882)	Overall (*N* = 43,150)
Sex				
Male	12,850 (43.5%)	5005 (51.5%)	1925 (49.6%)	19,780 (45.8%)
Female	16,693 (56.5%)	4720 (48.5%)	1957 (50.4%)	23,370 (54.2%)
Age groups (years)				
0–64	8488 (28.7%)	1185 (12.2%)	1211 (31.2%)	10,884 (25.2%)
65–84	13,610 (46.1%)	5574 (57.3%)	2125 (54.7%)	21,309 (49.4%)
≥ 85	7445 (25.2%)	2966 (30.5%)	546 (14.1%)	10,957 (25.4%)
Charlson comorbidity score				
0	6126 (20.7%)	0 (0%)	0 (0%)	6126 (14.2%)
1	5321 (18.0%)	0 (0%)	0 (0%)	5321 (12.3%)
2	4618 (15.6%)	2026 (20.8%)	1446 (37.2%)	8090 (18.7%)
3	3924 (13.3%)	2051 (21.1%)	871 (22.4%)	6846 (15.9%)
4	3011 (10.2%)	1664 (17.1%)	586 (15.1%)	5261 (12.2%)
5	6543 (22.1%)	3984 (41.0%)	979 (25.2%)	11,506 (26.7%)
Origin of CDI				
Community-acquired	2710 (9.2%)	318 (3.3%)	66 (1.7%)	3094 (7.2%)
Hospital-acquired	26,464 (89.6%)	9291 (95.5%)	3771 (97.1%)	39,526 (91.6%)
Unknown	369 (1.2%)	116 (1.2%)	45 (1.2%)	530 (1.2%)
Use of any antibiotics				
No	949 (3.2%)	201 (2.1%)	107 (2.8%)	1257 (2.9%)
Yes	28,594 (96.8%)	9524 (97.9%)	3775 (97.2%)	41,893 (97.1%)
Use of any PPI				
No	9168 (31.0%)	2242 (23.1%)	899 (23.2%)	12,309 (28.5%)
Yes	20,375 (69.0%)	7483 (76.9%)	2983 (76.8%)	30,841 (71.5%)
Use of any H2RA				
No	27,461 (93.0%)	8949 (92.0%)	3565 (91.8%)	39,975 (92.6%)
Yes	2082 (7.0%)	776 (8.0%)	317 (8.2%)	3175 (7.4%)
Use of any NSAIDS				
No	12,448 (42.1%)	3729 (38.3%)	1532 (39.5%)	17,709 (41.0%)
Yes	17,095 (57.9%)	5996 (61.7%)	2350 (60.5%)	25,441 (59.0%)
Use of any aspirin				
No	14,367 (48.6%)	4377 (45.0%)	2355 (60.7%)	21,099 (48.9%)
Yes	15,176 (51.4%)	5348 (55.0%)	1527 (39.3%)	22,051 (51.1%)
CDI recurrence				
No recurrence	24,574 (83.2%)	8002 (82.3%)	3323 (85.6%)	35,899 (83.2%)
Recurrence	4969 (16.8%)	1723 (17.7%)	559 (14.4%)	7251 (16.8%)
Death				
No death	25,603 (86.7%)	7700 (79.2%)	3184 (82.0%)	36,487 (84.6%)
Death	3940 (13.3%)	2025 (20.8%)	698 (18.0%)	6663 (15.4%)

H2RA, histamine-2 receptor antagonists; NSAIDs, non-steroidal anti-inflammatory drugs; PPI, proton pump inhibitors

### Observed proportion of CDI recurrence per cancer type

**Fig. 2:**
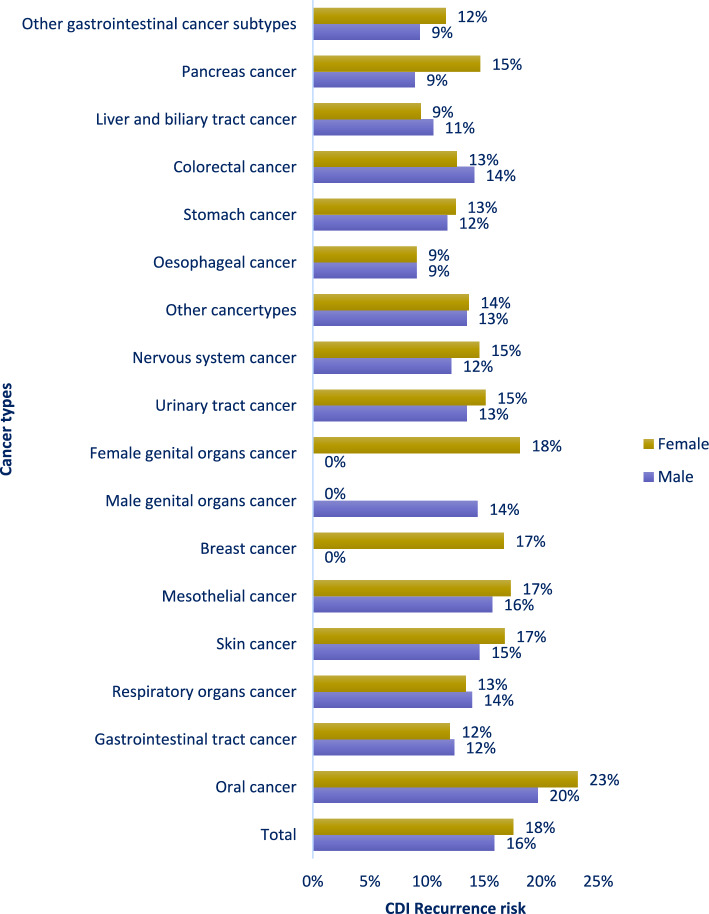
Observed proportion of recurrence for the different cancer types in individuals with *Clostridioides difficile* infection (CDI) and ongoing cancer (diagnosed < 1 year)

CDI recurrence varied across cancer types (Fig. 2, Supplementary Table 2). Among men, the highest proportions were observed in oral cancer (20%), mesothelial cancer (16%) and skin cancer (15%), while women show highest proportions in oral cancer (23%), female genital organs cancer (18%), breast cancer (17%), mesothelial cancer (17%) and skin cancer (17%). The proportion of CDI recurrence for the group of gastrointestinal cancer was 12% for both sexes.

### CDI recurrence

Individuals with ongoing cancer had a significantly lower odds of CDI recurrence (OR = 0.81, 95% CI 0.73–0.89) and significantly higher odds of death (OR = 1.58, 95% CI 1.43–1.74), compared to those without cancer. Among CDI-survivors, the odds of CDI recurrence remained significantly lower for individuals with ongoing cancer (OR = 0.84, 95% CI 0.76–0.94) compared to those without cancer (Table 2). Considering death as competing risk, individuals with ongoing cancer had a lower hazard of CDI recurrence (HR = 0.79, 95% CI 0.72–0.87) (Supplementary Table 3).

**Table 2:** The risk of recurrence and risk of death in all individuals with a *Clostridioides difficile* infection (CDI) in Sweden (2006–2019), calculated by multivariable logistic regression and expressed as odds ratio (OR) and 95% confidence interval (CI)

	No cancer	Ongoing cancer	Cancer history
Odds of recurrence	Reference	0.81 (0.73–0.89)	1.00 (0.93–1.06)
Odds of death	Reference	1.58 (1.43–1.74)	1.45 (1.36–1.55)
Odds of recurrence among CDI-survivors	Reference	0.84 (0.76–0.94)	1.04 (0.97–1.11)

*All results are adjusted for age, sex, origin (hospital-, or community acquired), chronic comorbidities and prescribed drug use*

The stratified analysis reveals varying odds of CDI recurrence associated with different cancer types among CDI-survivors compared to individuals without cancer (Table 3). Oral cancer shows a non-significant increased odds of CDI recurrence (OR = 1.37, 95% CI 0.87–2.08) among individuals with ongoing cancer. Mesothelial cancer shows a non-significant increased odds of CDI recurrence (OR 1.16, 95% CI 0.72–1.78) among individuals with ongoing cancer. Gastrointestinal tract cancer demonstrates a significantly reduced odds of CDI recurrence (OR = 0.69, 95% CI 0.55–0.86) among individuals with ongoing cancer. Skin cancer shows a significantly higher odds of CDI recurrence in individuals with cancer history (OR = 1.18, 95% CI 1.07–1.30). Esophageal cancer demonstrates a significantly reduced odds of CDI recurrence among CDI-survivors with ongoing cancer (OR = 0.46, 95% CI 0.18–0.99) and among CDI-survivors with cancer history (OR = 0.46, 95% CI 0.18–0.99). However, none of the other gastrointestinal subtypes, demonstrate statistically significant associations with CDI recurrence.

**Table 3:** Stratified analysis for the odds of CDI recurrence among CDI-survivors per cancer type, in all individuals with a Clostridioides difficile infection (CDI) in Sweden (2006–2019), expressed as odds ratios (OR) and 95% confidence intervals

Cancer type	No cancer	Ongoing cancer	Cancer history
Oral cancer	Reference	1.37 (0.87–2.08)	1.07 (0.78–1.45)
Gastrointestinal tract cancer	Reference	0.69 (0.55–0.86)	0.95 (0.82–1.10)
Respiratory organs cancer	Reference	0.84 (0.62–1.13)	1.11 (0.89–1.37)
Skin cancer	Reference	0.87 (0.67–1.12)	1.18 (1.07–1.30)
Mesothelial cancer	Reference	1.16 (0.72–1.78)	1.21 (0.86–1.67)
Breast cancer	Reference	0.91 (0.65–1.25)	0.99 (0.85–1.16)
Male genital organs cancer	Reference	0.80 (0.55–1.13)	0.97 (0.84–1.11)
Female genital organs cancer	Reference	0.98 (0.66–1.41)	1.02 (0.83–1.26)
Urinary tract cancer	Reference	0.82 (0.56–1.16)	1.04 (0.87–1.23)
Nervous system cancer	Reference	0.82 (0.44–1.43)	0.77 (0.48–1.19)
Other cancer	Reference	0.80 (0.70–0.92)	0.94 (0.85–1.04)
Esophageal cancer	Reference	0.38 (0.12–0.94)	0.46 (0.18–0.99)
Stomach cancer	Reference	0.68 (0.36–1.18)	0.72 (0.42–1.16)
Colorectal cancer	Reference	0.78 (0.58–1.02)	1.01 (0.85–1.20)
Liver and biliary tract cancer	Reference	0.54 (0.25–1.02)	0.94 (0.59–1.46)
Pancreas cancer	Reference	0.80 (0.44–1.34)	0.70 (0.40–1.13)
Other gastrointestinal-subtypes	Reference	0.60 (0.26–1.18)	0.88 (0.54–1.36)

*All results are adjusted for age, sex, origin (hospital-, or community acquired), chronic comorbidities and prescribed drug use*

**Fig. 3:**
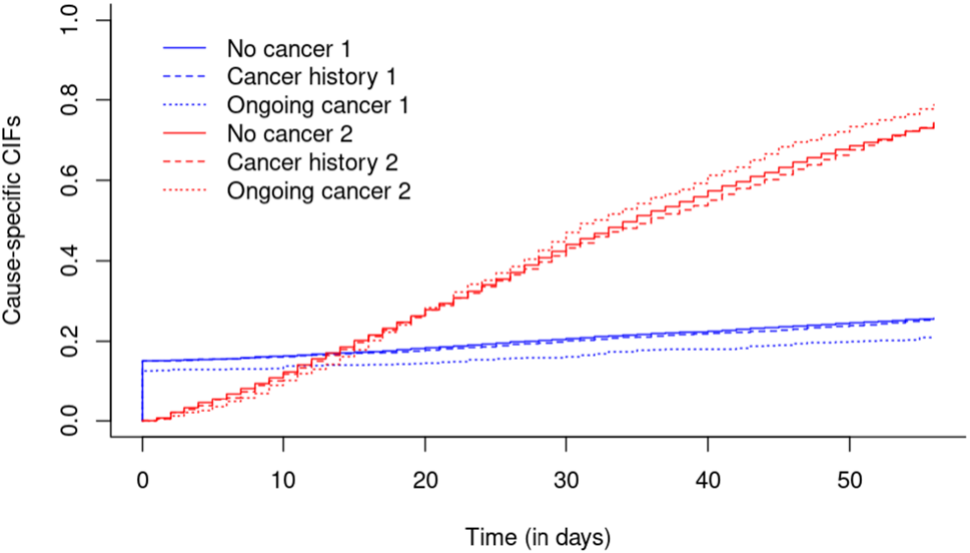
Cause specific cumulative incidence for all with or without (ongoing cancer)

The cause-specific cumulative incidence function (CIF) tailored to our study's competing risks data is visualized in Fig. 3.

### Effect of covariates on outcome

Significant associations were observed between different patient and CDI characteristics and the odds of CDI recurrence or death (Table 4). Women had a higher odds of CDI recurrence (OR = 1.12, 95% CI 1.07–1.18), but a lower odds of death (OR = 0.83, 95% CI 0.78–0.88), compared to men. The age group of 65–84 years had a higher odds of CDI recurrence (OR = 1.14, 95% CI 1.07–1.23), and death (OR = 4.02, 95% CI 3.59–4.51), compared to the 0–64 age group. Higher Charlson comorbidity scores were associated with increased odds of CDI recurrence and death. Hospital-acquired CDI was associated with a decreased odds of CDI recurrence (OR = 0.84, 95% CI 0.76–0.93) but a higher odds of death (OR = 5.74, 95% CI 4.33–7.80), compared to community-acquired CDI. The use of PPIs, NSAIDs, H2Ras and antibiotics was associated with an increased odds of CDI recurrence and decreased odds of death.

**Table 4:** *A*ssociation between patient characteristics and the odds of recurrence/death in all individuals with a *Clostridioides difficile* infection (CDI) in Sweden (2006–2019)*,* calculated by multivariable logistic regression and reported as odds ratios (ORs), 95% confidence intervals (CIs)

Characteristic	Odds of recurrence in all participants		Odds of recurrence in CDI-survivors		Odds of death in all participants	
	OR	95% CI	OR	95% CI	OR	95% CI
Cancer status						
No cancer	Ref		Ref		Ref	
Cancer history	1.00	0.93, 1.06	1.04	0.97, 1.11	1.45	1.36, 1.55
Ongoing cancer	0.81	0.73, 0.89	0.84	0.76, 0.94	1.58	1.43, 1.74
Sex						
Male	Ref		Ref		Ref	
Female	1.12	1.07, 1.18	1.12	1.06, 1.19	0.83	0.78, 0.88
Age groups (years)						
0–64	Ref		Ref		Ref	
65–84	1.14	1.07, 1.23	1.19	1.10, 1.27	4.02	3.59, 4.51
≥ 85	1.05	0.97, 1.14	1.16	1.06, 1.26	8.00	7.12, 9.00
Charlson comorbidity score						
0	Ref		Ref		Ref	
1	1.00	0.90, 1.11	1.00	0.90, 1.12	1.58	1.38, 1.81
2	1.06	0.96, 1.18	1.08	0.97, 1.20	1.68	1.48, 1.92
3	1.08	0.97, 1.20	1.09	0.98, 1.22	1.49	1.30, 1.70
4	1.14	1.02, 1.28	1.12	1.00, 1.27	1.49	1.30, 1.72
5	1.19	1.08, 1.32	1.20	1.08, 1.34	1.56	1.37, 1.78
Origin of CDI						
Community-acquired	Ref		Ref		Ref	
Hospital-acquired	0.84	0.76, 0.93	0.89	0.80, 0.99	5.74	4.33, 7.80
Unknown-acquired	1.30	1.04, 1.62	1.31	1.04, 1.63	1.77	1.02, 2.96
Use of any PPI						
No PPI	Ref		Ref		Ref	
Yes PPI	1.10	1.04, 1.17	1.08	1.01, 1.15	0.66	0.62, 0.70
Use of any NSAIDS						
No NSAIDS	Ref		Ref		Ref	
Yes NSAIDS	1.10	1.04, 1.16	1.07	1.01, 1.14	0.73	0.69, 0.77
Use of any H2RA						
No H2RA	Ref		Ref		Ref	
Yes H2RA	1.18	1.07, 1.29	1.17	1.06, 1.29	0.85	0.75, 0.96
Use of any aspirin						
No aspirin	Ref		Ref		Ref	
Yes aspirin	1.03	0.97, 1.09	1.02	0.96, 1.08	0.90	0.85, 0.96
Use of any antibiotics						
No antibiotics	Ref		Ref		Ref	
Yes antibiotics	2.01	1.65, 2.48	1.91	1.50, 2.48	0.30	0.26, 0.34

H2RA, histamine-2 receptor antagonists; NSAIDs, non-steroidal anti-inflammatory drugs; PPI, proton pump inhibitors

## Discussion

This study based on one of the largest nationwide cohorts of individuals with CDI, is to our knowledge, the first population study comparing CDI recurrence between different cancer types with the highest odds of occurrence in patients with oral cancer and lowest in those with esophageal cancer. Our study was not restricted to active malignancies as our individuals with cancer could have been hospitalized for non-cancer related indications, and our main analyzes included all individuals with a cancer diagnosis during the last year. This may partially explain the apparent overall lower odds of recurrence in cancer patients, even after correcting for mortality, compared to those without cancer. Yet, one in five with a recent cancer diagnosis (< 1 year) or cancer history died within 2 months after CDI; compared to only 13% of individuals without cancer, which may be primarily due to the cancer or to the CDI or a combination of both. It does suggest CDI is a serious complication for those with cancer, an important contributing factor to death of individuals with cancer, and a not-negligible competing risk in our models.

Our apparent less frequent recurrence compared to individuals without cancer may seem contra-intuitive and contrary to some previous studies which have suggested increased recurrence [50–52]. Yet, previous meta-analyzes did not find strong evidence for an association [14–16], and other studies show similar results [12, 53]. The inclusion of community-acquired CDI in our study probably made little difference, as 97% of those with ongoing cancer was deemed health-care acquired compared to 90% of those without cancer.

The strengths of our study are the large population-based cohort of 43,150 individuals, which enhances the strength of the findings. Secondly, the inclusion of multiple cancer types, among individuals with an ongoing cancer or cancer history, allowed for a comprehensive analysis of the association between cancer type and CDI recurrence. An important limitation is that we could not incorporate (neo-)adjuvant cancer treatment and in-hospital drug use (including antibiotic prophylaxis when e.g., cytotoxic chemotherapy is administered), as these were not recorded in the registries. We also lack information on frailty, body mass index, weight loss, smoking or other residual confounders. Due to the registry nature, no information was available on specific *C. difficile* strains, which may pose different risks of recurrence e.g., due to different antimicrobial resistance patterns [6, 24], and may have helped to distinguish between reinfection and relapse [34, 35]. Underreporting and underdiagnosing of CDI, may also have contributed to a selection bias toward more severe cases. Our study encountered the challenge of limited existing literature, posing complexities during the design. Nevertheless, to ensure methodological integrity and avoid data-driven exploration, we followed a prior developed study protocol. This protocol defined CDI recurrence within an 8-week timeframe after the initial episode, considering deaths within this period as competing events. For the logistic regressions, our results excluding deaths may have created a selection bias, yet leaving them in underestimated the odds of recurrence [54]. Although the cause-specific hazard model was utilized, the presence of right-censored observations, which may not precisely reflect true right censoring from a statistical standpoint, introduces the possibility that the impact of deaths on CDI recurrence might not be adequately captured. The classification of cancer into ongoing cancer and cancer history presented challenges due to the wide variation in the clinical course of different cancer types. We approached this issue by considering that patients who survived the initial cancer episode would likely undergo a period of intense treatment, including surgery, chemotherapy, and/or radiotherapy, lasting less than a year. We hypothesized that individuals would be at the highest risk of CDI and recurrence during this active treatment phase. However, it is important to recognize that for many patients, cancer has long-term health effects. They may not achieve remission, experience complications from cancer or its treatment, or have a reduced overall quality of life, all of which could potentially impact their risk of CDI and recurrence and survival.

Although it is interesting to compare all cancer types, this comes with major challenges including difficulties to compare cancer aggressiveness, staging and treatment—and even our cut-off of 12 months since cancer diagnosis is not sensitive enough to distinguish between actively treated malignancies and those cured or in remission. Intuitively, we expected a higher overall recurrence, and we do expect more recurrences if we could more clearly define those with active cancer, as they will be more commonly hospitalized and exposed to antibiotics. Although almost all individuals with cancer in our cohort were categorized under healthcare associated CDI (97.1%), it could be considered to subcategorise cancer by prior healthcare consumption (by for example number of hospitalisations, length of stay, specialist outpatient visits), types of cancer treatment or cancer staging—yet this is challenging for a cross-cancer overall analyzes.

We expected more frequent recurrences among individuals with gastrointestinal cancer because of gut microbiome disturbances, local (neo) adjuvant treatment etc., but found a lower odds; findings which have, to our knowledge, not been described in the literature, but are interesting from a mechanistic point of view, if this association is indeed true and not the result of residual confounding and/or biases. Yet, the highest, yet statistically unsignificant, odds of recurrence was found in oral and mesothelial cancer. This might suggest that anatomical location of the cancer may affect CDI infection or recurrence, beyond the immunocompromised state of the patient and/or different treatment practices including (neo-)adjuvant treatment, antibiotic prophylaxis, surgery and others. Interestingly, survivors with esophageal cancer exhibited a significantly reduced odds of CDI recurrence. Several explanations can be hypothesized, including different (neo-)adjuvant and other treatment regimens affecting the immunocompromised state of the patient and their gut and other microbiome compositions, and therefore risk of recurrence [55–58]. Gut biofilms may serve as a reservoir for *C. difficile* [59, 60], as well as other potential microbiome reservoirs such as the appendix [61, 62], resulting in relapses of the same infection, instead of a true recurrence [35, 63, 64]. As gastroesophageal reflux is a major risk factor for esophageal adenocarcinoma, increased stomach acid levels (gastric barrier function) [65], may create a challenging environment for *C. difficile*, and therefore hinder re-introduction of *C. difficile* spores—yet a large majority of individuals with oesophageal cancer is exposed to maintenance therapy with PPIs. The more frequent recurrence in oral cancer might suggest a role of the oral microbiome, and/or confounding effects of smoking and alcohol [66–69].

Our results may have raised more questions than answers on how *C. difficile* strains and spores may travel through the body and interact with different microbiome niches and cancer micro-environments; and also bring back the discussion if a recurrence is due to a relapse (same strain) or a new infection (new strain) [70–72]. Yet, we do want to stress the clinical implication that CDI is an important complication for individuals with a recent history of cancer but also for others, as mortality and of recurrence are common. There are possibilities for prevention of CDI and CDI recurrence, including an adapted, more personalized prescription regimen of antibiotics and PPIs [51, 73–76]. Besides the additional morbidity and mortality in patients with cancer, the occurrence of CDI in these patients also conflicts with their ongoing treatment plans by reducing or delaying cancer care [25, 52].

In conclusion, our study suggests that individuals with (a history of) cancer do not necessarily have more frequent CDI recurrences compared to those without cancer, yet CDI seems an important contributing factor to mortality, and therefore the potential consequences of recurrence may be large in individuals with cancer. The risk of CDI recurrence seems to vary among different cancer types which may bring interesting insights in how CDI recurs. These findings warrant further exploration particularly in individuals with cancer along the gastro-intestinal tract, to validate or disprove our results, and to also explore the effects of cancer staging and treatment.

### Supplementary Information

Below is the link to the electronic supplementary material.

Supplementary file1 (DOCX 69 KB)

## Data Availability

The data underlying this article were provided by Karolinska Institutet under license/by permission of the National Board of Health and Welfare who own the data. Data will be shared on request to the corresponding author on reasonable request after required approvals from the national Ethics Committee and National Board of Health and Welfare are obtained.
