# Supplementary material for: Clostridioides difficile recurrence in individuals with and without cancer: a Swedish population-based cohort study
Source: Infection. 2024 Feb 26;52(2):649–60. doi: 10.1007/s15010-024-02193-1 (PMC10954957; doi:10.1007/s15010-024-02193-1)
Supplement: Supplementary file 1 — Supplementary file1 (DOCX 69 KB) [file 15010_2024_2193_MOESM1_ESM.docx]

# Supplementary Materials

## **Supplementary Table 1**

## Definitions of cancer types by the International statistical Classification of Disease *-* 10th revision (ICD-10) codes*,* and prescribed drug use based on the Anatomical Therapeutic Chemical (ATC) classification of the World Health Organization.

| ICD-10 code | Category |
| --- | --- |
| C00 – C14 | Oral cancer |
| C15 – C26 | Gastrointestinal tract cancer |
| C30 – C39 | Respiratory organs cancer |
| C43 | Skin cancer - melanoma |
| C45 – C49 | Mesothelial cancer |
| C50 – C50 | Breast cancer |
| C51 – C58 | Female genital organs cancer |
| C60 – C63 | Male genital organs cancer |
| C64 – C68 | Urinary tract cancer |
| C69 – C72 | Nervous system cancer |
| C40 – 41  + C73 – C75  + C76 – C80  + C81 – C96  + C97 | Other types: Bone and articular cartilage cancer, ill-defined, secondary and unspecified sites cancer, thyroid and other endocrine glands cancer, Haematological cancer (lymphoid, hematopoietic and related tissue), independent (primary) multiple sites cancer |
| C15 | Esophageal cancer |
| C16 | Stomach cancer |
| C18 – C20 | Colorectal cancer: colon cancer, rectosigmoid junction cancer, rectum cancer |
| C22 - 24 | Liver and biliary tract cancer: liver and intrahepatic bile ducts cancer, gallbladder cancer, other unspecified parts of biliary tract cancer |
| C25 | Pancreas cancer |
| C17 + C21  + C26 | Other Gastrointestinal subtypes cancer: small intestine cancer, anus and anal canal cancer, ill-defined digestive organs cancer |
| ATC code | Category |
| J01 | Systemic antibiotic use |
| B01AC06  + N02BA | Aspirin |
| A02BA | H2-receptor antagonists (H2RA) |
| A02BC | Proton pump inhibitor (PPI) |
| M01A | Non-steroidal anti-inflammatory drugs (NSAIDS) |

**Supplementary Table 2 Descriptive statistics of individuals with ongoing cancer and cancer history, by cancer location**

|  | | | | | | |
| --- | --- | --- | --- | --- | --- | --- |
|  | **Male** | | **Female** | | **Overall** | |
|  | **No recurrence** | **Recurrence** | **No recurrence** | **Recurrence** | **No recurrence** | **Recurrence** |
|  | **(N=16635)** | **(N=3145)** | **(N=19264)** | **(N=4106)** | **(N=35899)** | **(N=7251)** |
| **Oral cancer** |  |  |  |  |  |  |
| Cancer history | 133 (0.8%) | 29 (0.9%) | 115 (0.6%) | 32 (0.8%) | 248 (0.7%) | 61 (0.8%) |
| Ongoing cancer | 53 (0.3%) | 13 (0.4%) | 53 (0.3%) | 16 (0.4%) | 106 (0.3%) | 29 (0.4%) |
| **Gastrointestinal tract cancer** |  |  |  |  |  |  |
| Cancer history | 733 (4.4%) | 131 (4.2%) | 597 (3.1%) | 133 (3.2%) | 1330 (3.7%) | 264 (3.6%) |
| Ongoing cancer | 411 (2.5%) | 58 (1.8%) | 359 (1.9%) | 49 (1.2%) | 770 (2.1%) | 107 (1.5%) |
| **Respiratory organs cancer** |  |  |  |  |  |  |
| Cancer history | 276 (1.7%) | 53 (1.7%) | 290 (1.5%) | 77 (1.9%) | 566 (1.6%) | 130 (1.8%) |
| Ongoing cancer | 179 (1.1%) | 29 (0.9%) | 220 (1.1%) | 34 (0.8%) | 399 (1.1%) | 63 (0.9%) |
| **Skin cancer** |  |  |  |  |  |  |
| Cancer history | 1458 (8.8%) | 339 (10.8%) | 1412 (7.3%) | 358 (8.7%) | 2870 (8.0%) | 697 (9.6%) |
| Ongoing cancer | 228 (1.4%) | 39 (1.2%) | 228 (1.2%) | 46 (1.1%) | 456 (1.3%) | 85 (1.2%) |
| **Mesothelial cancer** |  |  |  |  |  |  |
| Cancer history | 80 (0.5%) | 17 (0.5%) | 122 (0.6%) | 33 (0.8%) | 202 (0.6%) | 50 (0.7%) |
| Ongoing cancer | 59 (0.4%) | 11 (0.3%) | 62 (0.3%) | 13 (0.3%) | 121 (0.3%) | 24 (0.3%) |
| **Breast cancer** |  |  |  |  |  |  |
| Cancer history | 26 (0.2%) | 5 (0.2%) | 1005 (5.2%) | 221 (5.4%) | 1031 (2.9%) | 226 (3.1%) |
| Ongoing cancer | 3 (0.0%) | 0 (0%) | 229 (1.2%) | 46 (1.1%) | 232 (0.6%) | 46 (0.6%) |
| **Male genital organs cancer** |  |  |  |  |  |  |
| Cancer history | 1666 (10.0%) | 313 (10.0%) | 1 (0.0%) | 0 (0%) | 1667 (4.6%) | 313 (4.3%) |
| Ongoing cancer | 249 (1.5%) | 42 (1.3%) | 0 (0%) | 0 (0%) | 249 (0.7%) | 42 (0.6%) |
| **Female genital organs cancer** |  |  |  |  |  |  |
| Cancer history | 1 (0.0%) | 0 (0%) | 581 (3.0%) | 131 (3.2%) | 582 (1.6%) | 131 (1.8%) |
| Ongoing cancer | 0 (0%) | 0 (0%) | 185 (1.0%) | 41 (1.0%) | 185 (0.5%) | 41 (0.6%) |
| **Urinary tract cancer** |  |  |  |  |  |  |
| Cancer history | 619 (3.7%) | 118 (3.8%) | 272 (1.4%) | 72 (1.8%) | 891 (2.5%) | 190 (2.6%) |
| Ongoing cancer | 154 (0.9%) | 24 (0.8%) | 101 (0.5%) | 18 (0.4%) | 255 (0.7%) | 42 (0.6%) |
| **Nervous system cancer** |  |  |  |  |  |  |
| Cancer history | 86 (0.5%) | 11 (0.3%) | 73 (0.4%) | 13 (0.3%) | 159 (0.4%) | 24 (0.3%) |
| Ongoing cancer | 58 (0.3%) | 8 (0.3%) | 41 (0.2%) | 7 (0.2%) | 99 (0.3%) | 15 (0.2%) |
|  |  |  |  |  |  |  |
| **Other cancertypes** |  |  |  |  |  |  |
| Cancer history | 1706 (10.3%) | 292 (9.3%) | 1556 (8.1%) | 344 (8.4%) | 3262 (9.1%) | 636 (8.8%) |
| Ongoing cancer | 1029 (6.2%) | 160 (5.1%) | 1068 (5.5%) | 169 (4.1%) | 2097 (5.8%) | 329 (4.5%) |
| **Gastro-intestinal cancer - subtypes** | | | | | | |
| **Oesophagus cancer** |  |  |  |  |  |  |
| Cancer history | 53 (0.3%) | 4 (0.1%) | 19 (0.1%) | 4 (0.1%) | 72 (0.2%) | 8 (0.1%) |
| Ongoing cancer | 40 (0.2%) | 4 (0.1%) | 20 (0.1%) | 2 (0.0%) | 60 (0.2%) | 6 (0.1%) |
| **Stomach cancer** |  |  |  |  |  |  |
| Cancer history | 104 (0.6%) | 13 (0.4%) | 40 (0.2%) | 7 (0.2%) | 144 (0.4%) | 20 (0.3%) |
| Ongoing cancer | 60 (0.4%) | 8 (0.3%) | 42 (0.2%) | 6 (0.1%) | 102 (0.3%) | 14 (0.2%) |
| **Colorectal cancer** |  |  |  |  |  |  |
| Cancer history | 458 (2.8%) | 97 (3.1%) | 417 (2.2%) | 91 (2.2%) | 875 (2.4%) | 188 (2.6%) |
| Ongoing cancer | 219 (1.3%) | 36 (1.1%) | 187 (1.0%) | 27 (0.7%) | 406 (1.1%) | 63 (0.9%) |
| **Liver and biliary tract cancer** |  |  |  |  |  |  |
| Cancer history | 85 (0.5%) | 11 (0.3%) | 53 (0.3%) | 14 (0.3%) | 138 (0.4%) | 25 (0.3%) |
| Ongoing cancer | 51 (0.3%) | 6 (0.2%) | 48 (0.2%) | 5 (0.1%) | 99 (0.3%) | 11 (0.2%) |
| **Pancreas cancer** |  |  |  |  |  |  |
| Cancer history | 68 (0.4%) | 6 (0.2%) | 56 (0.3%) | 12 (0.3%) | 124 (0.3%) | 18 (0.2%) |
| Ongoing cancer | 51 (0.3%) | 5 (0.2%) | 64 (0.3%) | 11 (0.3%) | 115 (0.3%) | 16 (0.2%) |
| **Other** |  |  |  |  |  |  |
| Cancer history | 66 (0.4%) | 7 (0.2%) | 69 (0.4%) | 16 (0.4%) | 135 (0.4%) | 23 (0.3%) |
| Ongoing cancer | 29 (0.2%) | 3 (0.1%) | 38 (0.2%) | 5 (0.1%) | 67 (0.2%) | 8 (0.1%) |

**Supplementary Table 3: The association between various patient characteristics and the hazard of recurrence/death in all individuals with a Clostridioides difficile infection (CDI) in Sweden (2006-2019), calculated by multivariable cause-specific hazards regression, and expressed as hazard ratios (HRs) and 95% confidence intervals (CIs).**

| Characteristic | HR | 95% CI |
| --- | --- | --- |
| Cancer status |  |  |
| No cancer | Ref | Ref |
| Cancer history | 0.95 | 0.89, 1.00 |
| Ongoing cancer | 0.79 | 0.72, 0.87 |
| Sex |  |  |
| Male | Ref | Ref |
| Female | 1.12 | 1.07, 1.18 |
| Age groups |  |  |
| 1-64 | Ref | Ref |
| 65-84 | 1.06 | 0.99, 1.13 |
| ≥85 | 0.91 | 0.84, 0.98 |
| Charlson comorbidity score |  |  |
| 0 | Ref | Ref |
| 1 | 0.97 | 0.88, 1.07 |
| 2 | 1.01 | 0.93, 1.11 |
| 3 | 1.04 | 0.94, 1.14 |
| 4 | 1.09 | 0.98, 1.20 |
| 5 | 1.13 | 1.03, 1.24 |
| Origin of CDI |  |  |
| Community-acquired | Ref | Ref |
| Hospital-acquired | 0.82 | 0.75, 0.90 |
| Unknown | 1.27 | 1.04, 1.54 |
| Use of any antibiotics |  |  |
| No antibiotics | Ref | Ref |
| Yes antibiotics | 2.15 | 1.77, 2.61 |
| Use of any PPI |  |  |
| No PPI | Ref | Ref |
| Yes PPI | 1.14 | 1.07, 1.20 |
| Use of any NSAIDS |  |  |
| No NSAIDS | Ref | Ref |
| Yes NSAIDS | 1.12 | 1.06, 1.17 |
| Use of any H2RA |  |  |
| No H2RA | Ref | Ref |
| Yes H2RA | 1.16 | 1.07, 1.26 |
| Use of any aspirin |  |  |
| No aspirin | Ref | Ref |
| Yes aspirin | 1.03 | 0.98, 1.09 |

H2RA, histamine-2 receptor antagonists; NSAIDs, non-steroidal anti-inflammatory drugs; PPI, proton pump inhibitors
